# An atypical form of 60S ribosomal subunit in Diamond-Blackfan anemia linked to *RPL17* variants

**DOI:** 10.1172/jci.insight.172475

**Published:** 2024-08-01

**Authors:** Florence Fellmann, Carol Saunders, Marie-Françoise O’Donohue, David W. Reid, Kelsey A. McFadden, Nathalie Montel-Lehry, Cong Yu, Mingyan Fang, Jianguo Zhang, Beryl Royer-Bertrand, Pietro Farinelli, Narjesse Karboul, Jason R. Willer, Lorraine Fievet, Zahurul Alam Bhuiyan, Alissa L.W. Kleinhenz, Julie Jadeau, Joy Fulbright, Carlo Rivolta, Raffaele Renella, Nicholas Katsanis, Jacques S. Beckmann, Christopher V. Nicchitta, Lydie Da Costa, Erica E. Davis, Pierre-Emmanuel Gleizes

**Affiliations:** 1 The ColLaboratory, University of Lausanne, Lausanne, Switzerland.; 2 Service of Medical Genetics, University Hospital Lausanne (CHUV), Lausanne, Switzerland.; 3 University of Missouri Kansas City, School of Medicine, Kansas City, Missouri, USA.; 4 Department of Pathology and Laboratory Medicine, Children’s Mercy Hospital, Kansas City, Missouri, USA.; 5 MCD, Centre de Biologie Intégrative, Université de Toulouse, CNRS, UPS, Toulouse, France.; 6 Department of Biochemistry, Duke University Medical Center, Durham, North Carolina, USA.; 7 Center for Human Disease Modeling, Duke University Medical Center, Durham, North Carolina, USA.; 8 BGI-Shenzhen, Shenzhen, China.; 9 Department of Medical Genetics, University of Lausanne, Lausanne, Switzerland.; 10 UMR 1149 INSERM, Faculté de Médecine Site Bichat, Paris, France.; 11 Division of Hematology/Oncology, Children’s Mercy Hospital and Clinics, Kansas City, Missouri, USA.; 12 Division of Pediatrics, University Hospital Lausanne (CHUV), Lausanne, Switzerland.; 13 Clinical Bioinformatics, SIB Swiss Institute of Bioinformatics, Lausanne, Switzerland.; 14 Department of Cell Biology, Duke University Medical Center, Durham, North Carolina, USA.; 15 AP-HP, Service d’Hématologie Biologique, Hôpital Robert Debré, Paris, France.; 16 Université Paris Cité, Paris, France.; 17 Hematim EA4666, CURS, CHU Amiens, Amiens, France.; 18 LABEX GR-EX, Paris, France.; 19 Stanley Manne Children’s Research Institute, Ann & Robert H. Lurie Children’s Hospital of Chicago, Chicago, Illinois, USA.; 20 Departments of Pediatrics and Cell and Developmental Biology, Feinberg School of Medicine, Northwestern University, Chicago, Illinois, USA.

**Keywords:** Genetics, Hematology, Bone marrow, RNA processing, Translation

## Abstract

Diamond-Blackfan anemia syndrome (DBA) is a ribosomopathy associated with loss-of-function variants in more than 20 ribosomal protein (RP) genes. Here, we report the genetic, functional, and biochemical dissection of 2 multigenerational pedigrees with variants in *RPL17*, a large ribosomal subunit protein–encoding gene. Affected individuals had clinical features and erythroid proliferation defects consistent with DBA. Further, RPL17/uL22 depletion resulted in anemia and micrognathia in zebrafish larvae, and in vivo complementation studies indicated that *RPL17* variants were pathogenic. Lymphoblastoid cell lines (LCLs) derived from patients displayed a ribosomal RNA maturation defect reflecting haploinsufficiency of *RPL17*. The proteins encoded by *RPL17* variants were not incorporated into ribosomes, but 10%–20% of 60S ribosomal subunits contained a short form of 5.8S rRNA (5.8S_C_), a species that is marginal in normal cells. These atypical 60S subunits were actively engaged in translation. Ribosome profiling showed changes of the translational profile, but those are similar to LCLs bearing *RPS19* variants. These results link an additional RP gene to DBA. They show that ribosomes can be modified substantially by *RPL17* haploinsufficiency but support the paradigm that translation alterations in DBA are primarily related to insufficient ribosome production rather than to changes in ribosome structure or composition.

## Abstract 2

Analysis of two families, including a multi-generational pedigree, link Diamond-Blackfan anemia to variants of the ribosomal protein gene *RPL17* and reveal unusual ribosomal RNA heterogeneity.

## Introduction

Diamond-Blackfan Anemia syndrome (DBA) is a rare autosomal dominant syndrome characterized by congenital aplastic anemia, usually macrocytic, coupled with variable craniofacial, digit, cardiac, and urogenital defects (1–3). Approximately 1 in 100,000 to 1 in 200,000 live births are affected by the disorder, with the predominant hematopoietic phenotypes evident within the first year of life. Most DBA cases can be attributed to haploinsufficiency of more than 20 different ribosomal proteins (RPs), making DBA part of a growing class of disorders known as ribosomopathies (4–7).

The DBA anemia phenotype is driven by proliferation arrest in erythroid progenitors, resulting in hypocellular bone marrow and reduced levels of mature erythroid cells (8). Accumulation of ribosomal RNA (rRNA) precursors is a hallmark of cells from patients with DBA, reflecting the contribution of RPs to rRNA maturation (7, 9–14). Such defects in ribosome biogenesis constitute a “nucleolar stress” (15), which triggers different response pathways, including activation of p53. In current paradigms, defective assembly of the ribosomal subunit precursors (preribosomes) leads to increased levels of free 5S RNP, a ribonucleoprotein particle incorporated into the large ribosomal subunit and made of the 5S rRNA, RPL5/uL18, and RPL11/uL5. The free 5S RNP then binds and deactivates HDM2, a ubiquitin ligase that negatively controls p53 levels (16–20). The resulting stabilization of p53 may contribute to the pathophysiology of ribosomopathies, as shown in various cellular and animal models whose phenotype is rescued, at least partially, in a p53 null background (21–23). Other nucleolar stress response pathways may also be involved, but their mechanisms remain incompletely understood (24). Contribution of apoptosis to the erythroid progenitor proliferation defect was found to vary according to the affected gene (25, 26).

Ribosome biogenesis defects are also expected to alter the number, and, potentially, the structure, of ribosomes in the cell, thereby affecting the regulation of protein synthesis. Such a defect could impair synthesis of master regulator proteins involved in specific physiological processes. Hence, it has been proposed that erythroid specificity of DBA may be conferred by reduced synthesis and stability of GATA1, a transcription factor that is essential for erythroid maturation, either due to reduced translation of *GATA1* mRNA (27) or to lack of its chaperone HSP70, which normally protects GATA1 from caspase-3 cleavage during terminal erythroid differentiation upon EPO stimulation (28).

Explaining the tissue specificity of ribosomopathies remains a central question in the field. Although initially attributed to DBA exclusively, constitutional RP variants have been linked recently to disease phenotypes that do not involve bone marrow failure. For example, haploinsufficiency of the large ribosomal subunit protein gene *RPL10* leads to neurodevelopmental deficits and intellectual disability (29, 30). Similarly, pathogenic variants in *RPS23* were found in individuals displaying microcephaly and overlapping dysmorphic features (31), likely due to defects in translation accuracy. More recently, *RPL13* variants were found to cause spondyloepimetaphyseal dysplasia with severe short stature (32, 33).

Here, we report 2 pedigrees with individuals bearing variants in *RPL17* and exhibiting erythroid maturation failure and skeletal development defects characteristic of DBA. Modeling of *rpl17* deficiency in zebrafish recapitulated the major clinical features of the disorder. Unlike any other case of DBA described to date, *RPL17* haploinsufficiency in patient-derived LCLs generates ribosomes containing a very short form of 5.8S rRNA. Despite this alternative population of ribosomes, LCLs with *RPL17* variants displayed a translational profile similar to LCLs carrying other RP gene variants, supporting the view that DBA is predominantly related to insufficient amounts of ribosomes rather than specific changes in ribosome structure or composition.

## Results

### Pathogenic variants in RPL17 cause DBA.

We identified rare *RPL17* variants in 2 pedigrees presenting with clinically overlapping phenotypes and a cohort of 55 DBA cases.

**Figure 1:**
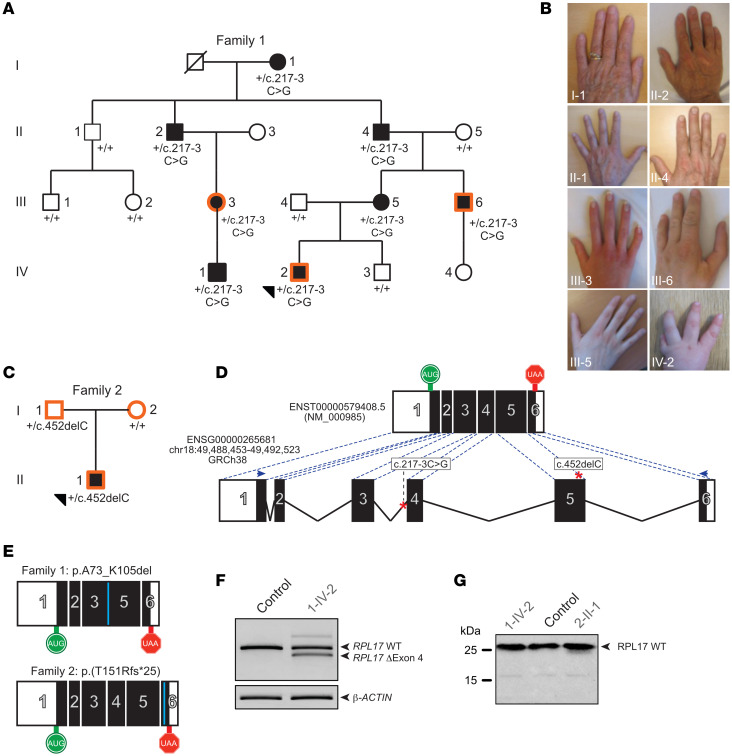
Pathogenic variants in *RPL17* cause Diamond-Blackfan anemia. (**A**) 4-generation Swiss pedigree (family 1) harboring a heterozygous c.217-3C>G variant in *RPL17*. Filled shapes, affected individuals; unfilled shapes, unaffected individuals; orange outline of shapes, exome sequencing was performed on DNA from that individual; “+”, WT allele; black triangle, index case. Individuals for whom no genotype is indicated means that a DNA sample was unavailable. (**B**) *RPL17* mutation carriers of family 1 display digit phenotypes including fifth finger hypoplasia (1-I-1, 1-II-2, 1-III-3, 1-II-1, 1-II-4, and 1-III-5) and absent thumbs (1-III-6 and 1-IV-2). (**C**) Family 2 harbors a heterozygous c.452delC *RPL17* variant and displays incomplete penetrance. Colors and symbols are the same as indicated in panel A. (**D**) Schematic of the WT human *RPL17* locus (bottom) and mRNA (top). Black boxes, coding exons; white boxes, untranslated regions; solid black lines, introns; green “AUG”, start codon; red “UAA”, stop codon; and variants identified in this study, red (pathogenic) or green (benign) asterisks. (**E**) *RPL17* variants identified in each of families 1 and 2 produce aberrant mRNA transcripts; blue bars in the schematic highlight the site of alteration. (**F**) RT-PCR products separated on agarose gel indicating that c.217C>G results in an in-frame deletion of exon 4. (**G**) Immunoblot of RPL17 protein (21.4 kDa) in LCLs derived from cases (gray) and unaffected individuals (black bold) from families 1 and 2.

Family 1 was ascertained through a male infant who was delivered at 41 weeks 4 days gestation and was at or below the 10th centile for weight, length, and head circumference (family 1; Figure 1A; Supplemental Case Reports; supplemental material available online with this article; https://doi.org/10.1172/jci.insight.172475DS1). Physical examination revealed a series of craniofacial anomalies including low-set ears; microretrognathism; glossoptosis; high arched palate; posterior median cleft palate; hypoplasia of the ascending mandibular branches; and a bilateral pretragian fistula (Supplemental Figure 1, A and B). Further, he displayed left thumb agenesis (Figure 1B, IV-2); right floating thumb; hypoplastic fifth fingers; and bilateral clinodactyly of the fifth toes. Consequently, his facial and digit malformations (Table 1) have mandated multiple corrective surgeries.

Family 1’s medical history suggested an autosomal dominant trait (Figure 1A, Table 1, and Supplemental case report). The mother of the index case (1-III-5) presented at birth with mild facial features including microretrognathism and a partial cleft palate that required surgical repair. Additionally, she had low hemoglobin levels (8.5 g/dL; Normal: 10.5–13.5 g/dL) as a neonate, and hematological follow-up throughout her lifetime has shown moderate, but persistent anemia. The maternal uncle of the index case (1-III-6) presented with bilateral absent thumbs at birth and was followed during infancy for moderate anemia that did not necessitate treatment. In total, 8 members of this 4-generation nonconsanguineous pedigree of northern European descent (Swiss) displayed variable congenital anomalies of the hands (absent thumbs: 2 of 8; fifth finger hypoplasia: 5 of 8; Figure 1B) or facial anomalies (7 of 8), with a range of hematopoietic conditions (anemia: 2 of 8; neutropenia: 2 of 8; anemia-neutropenia-thrombocytopenia: 1 of 8: neutropenia-thrombocytopenia-leukemia (AML): 1 of 8; Table 1).

Exome sequencing (ES) for 3 affected individuals (1-III-3, 1-III-6, and 1-IV-2) in family 1 was completed on DNA from peripheral blood using an Agilent capture library and Illumina sequencing to a mean target coverage ranging from 92–98 × (Supplemental Table 1). Assuming autosomal dominant inheritance, an allele frequency cutoff of 1% was used to identify heterozygous changes predicted to affect protein function or mRNA splicing and shared across the 3 individuals (Supplemental Table 2). After excluding variants in known genes for lack of phenotypic overlap, we retained an intronic variant (NM_000985.5: c.217-3C>G; p.?) near the exon 4 splice acceptor site of *RPL17*, encoding a component of the 60S ribosomal subunit. This variant was present in all 8 affected family members, but absent from all healthy individuals tested (Figure 1A).

RT-PCR studies on extracted total RNA from lymphoblastoid cell lines (LCLs) derived from the index case showed 2 different amplification products: the full-length transcript corresponding to the WT allele, present in lower amounts than in control cells, and a smaller variant, which lacks exon 4 (p.A73_K105del; Figure 1, D–F). The 33 amino acids encoded by exon 4 make a long α-helix within the structured core of RPL17, whose absence is likely to destabilize the protein. Accordingly, immunoblotting of the protein lysate from LCLs from the proband with an antibody developed against the N-terminus of the RPL17 revealed the full-length protein (184 amino acids), but not the predicted truncated form (1-IV-2, in Figure 1G). These data indicate that the *RPL17* c.217-3C>G variant causes skipping of exon 4 and affects production or stability of the protein, leading to haploinsufficiency of *RPL17*, which could cause the DBA phenotype observed in family 1.

The patient in family 2 (individual 2-II-1 in Figure 1C) presented during infancy with recurrent emesis; he was below the third centile for height and weight, displayed mild dysmorphic features, and complete blood count (CBC) showed macrocytic anemia, neutropenia, and mild thrombocytopenia (Supplemental case report; Table 1). His parents, of Northern European descent, were reportedly healthy. Following multiple negative clinical test results, trio ES on DNA isolated from peripheral blood was performed on an Illumina HiSeq2500 sequencer. We generated 2 × 125 bp sequenced paired-end reads to a mean coverage of 137× across the trio (Supplemental Table 1). We used SSAGA (Symptom and Sign Assisted Genome Analysis) (34) to map case 2-II-1’s clinical features (failure to thrive, intrauterine growth retardation, pancytopenia, short stature, wide intermammillary distance, eczema, bone marrow hypocellularity, and asthma) against 3,813 OMIM genes to nominate a total of 95 candidate variants for genetic analysis following all possible inheritance paradigms, none of which overlapped with the case phenotype (Supplemental Table 3). Genes of uncertain significance were investigated by removing the OMIM filter revealing a paternally inherited variant in *RPL17*, c.452delC; p.(T151Rfs*25), which is absent from population databases of healthy controls (ExAC and gnomAD; Figure 1, D and E). Full-length RPL17 protein, but not a truncated polypeptide, was detectable in protein lysate from LCLs from the index case in family 2 (2-II-1; Figure 1G). Thus, this variant also results in *RPL17* haploinsufficiency.

To identify additional cases with *RPL17* variants, we sequenced the coding region and splice junctions of *RPL17* using bidirectional Sanger sequencing in a cohort of 55 individuals fulfilling clinical diagnostic criteria for DBA. A heterozygous variant, c.167A>T; p.Q56L, was identified in a single individual. This variant is present in gnomAD (8 of 280,774 total alleles; 3 controls), and is predicted by in silico tools to be benign (PolyPhen-2 score of 0.002).

### Individuals with RPL17 variants display erythropoiesis defects.

**Figure 2:**
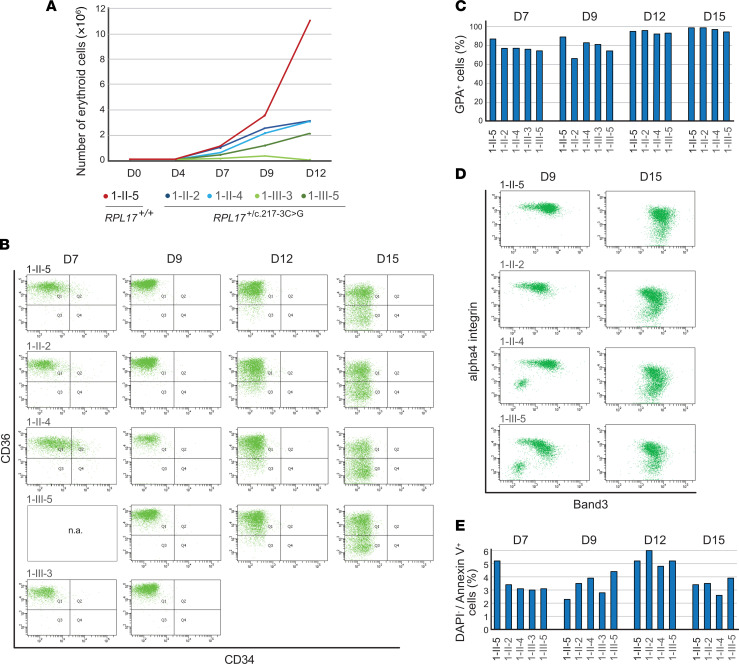
Characterization of erythroid maturation defects in family 1 from cells cultured in vitro. (**A**) Cell growth curves during erythroid cell differentiation; 1-II-2, 1-II-4, 1-III-3, and 1-III-5 harbor the *RPL17* c.217-3C>G variant; 1-II-5 is a healthy control (married-in spouse); D, day of culture. (**B**) Time course of CD34 and CD36 labelling. FACS analysis at D7 showed no consistent change in the percentage of BFU-e (CD34^+^/ CD36^–^) or CFU-e (CD34^–^/ CD36^+^) progenitor cells with *RPL17* variants. The gradual loss of CD36 labelling from D12 to D15, indicative of terminal differentiation stages, occurred with similar kinetics in cells from both affected and unaffected individuals. Profound cell death of sample 1-III-3 prohibited study beyond D9. (**C**) Quantification of GPA^+^ cells (erythroid specific marker) by FACS from D7 to D15. (**D**) Erythropoietic differentiation was assessed by co-detection of α4-integrin and Band3 in GPA^+^ cells. Increase in Band3 labelling, a marker of terminal erythroid cells, is paralleled by a decrease of α4-integrin during erythroid differentiation (35). (**E**) Quantification of the early apoptotic marker, Annexin V from D7 to D15 shows no significant apoptosis in patient-derived cells compared with control cells.

Terminal erythroid differentiation was studied on circulating primitive human CD34^+^ hematopoietic stem cells isolated from 4 affected individuals of family 1 (1-II-2, 1-II-4, 1-III-3, and 1-III-5) and a healthy intrafamilial control (1-II-5). All 4 affected members exhibited a defect in erythroid proliferation compared with the healthy control, with varying levels of hypoplasia in cultures monitored from day (D)0 to D12. Erythroid cells derived from individuals 1-II-2 and 1-II-4 grew similarly at an intermediate culture growth rate, but at a higher proliferation rate than the cells from individuals 1-III-5 and 1-III-3 (Figure 2A). Erythroid cells from 1-III-3 displayed the lowest proliferation rate and most of the cells died after D9.

Cells from individuals with DBA cultured in vitro were shown to display hallmark changes in membrane surface markers of human erythroid differentiation (25). When compared to nonaffected cells, *RPL17*-mutated cells at D7 showed no consistent changes in the fraction of cells positive for CD34, a marker of hematopoietic stem cells and progenitors until burst-forming unit–erythroid (BFU-e), or CD36, a marker that typically displays from the colony-forming units–erythroid (CFU-e) and the complete terminal differentiation (Figure 2B and Supplemental Figure 2). Cells positive for Glycophorin A (GPA^+^), a specific erythroid precursor, and gated cell marker, were 8%–25% less abundant in *RPL17^+/mut^* cells at D7 and D9 compared with the control, but this difference resolved by D12 (Figure 2C). Consistently, codetection of α4-integrin and Band3 (35) showed a similar change in the labeling patterns between D9 and D15 in cells from patients 1-II-2, 1-II-4, and 1-III-5 when compared with control cells, indicating that these cells progressed through differentiation (Figure 2D). In parallel, gradual disappearance of CD36 labelling, which occurs in terminal differentiation stages, was observed with similar kinetics in all cultures at D12 and D15. We observed no increase of the percentage of DAPI^–^/Annexin V^+^ erythroid cells in samples from all cases bearing the *RPL17* variant (Figure 2E). While these results suggest that apoptosis is not strongly induced in these cells, studies of additional samples will be required to fully characterize what prevents proliferation in *RPL17*^+/mut^ erythroid progenitors. Together, our results confirmed that individuals harboring the *RPL17* c.217-3C>G variant display altered erythroid proliferation, consistent with a haploinsufficiency paradigm characteristic of other RP genes mutated in DBA.

### Zebrafish models of RPL17 depletion recapitulate hallmark phenotypes of DBA.

We sought direct evidence in support of a causal link between *RPL17* genotype and clinical phenotype using zebrafish models. The developmental processes underlying hematopoiesis and establishment of the pharyngeal skeleton are conserved, in large part, between humans and zebrafish (36, 37), and zebrafish models for at least 8 ribosomal genes implicated in DBA have recapitulated both the hallmark anemia and craniofacial patterning defects (38–45). We identified the sole ortholog of *RPL17* in the *D*. *rerio* genome (95% identity, 97% similarity versus human protein) and targeted it with CRISPR/Cas9 genome editing using a single guide (sg)RNA targeting the coding region of *rpl17* exon 6 along with Cas9 protein in single-cell–stage zebrafish embryos (Supplemental Figure 3A). Founder generation (F0) mutant embryos had an estimated mosaicism of 68% based on cloning and sequencing of PCR products from 6 injected larvae (Supplemental Figure 3, B and C).

**Figure 3:**
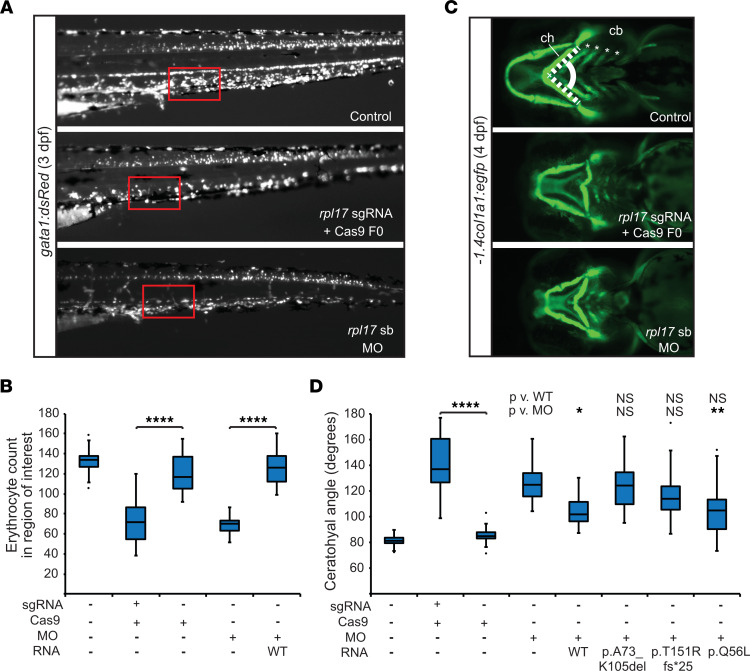
Zebrafish models of *rpl17* ablation display anemia and craniofacial patterning defects. (**A**) Representative lateral views of *gata1:dsRed* larvae imaged at 3 days postfertilization (dpf). Fluorescent signal, indicative of erythroid precursors and erythrocytes, was quantified in a consistently sized region of interest (red box) located posterior of the cloaca on the ventral side of controls, F0 mutants, and MO-injected larvae. Anterior, left; posterior, right. (**B**) Quantification of dsRed+ cells (erythroid cells) in the region of interest (see panel **A**) in F0 mutant or morphant larvae at 3 dpf. *n* = 20–40 larvae/batch, repeated at least twice. (**C**) Representative ventral views of *–1.4col1a1:egfp* larvae imaged at 4 dpf. Fluorescent signal was assessed for cartilage patterning defects by measuring the angle of the ceratohyal (ch) cartilage (dashed lines). Ceratobranchial (cb) arches were also dysplastic and reduced in number compared with controls. Anterior, left; posterior, right. (**D**) Quantification of the ch angle in F0 mutant and morphant larvae at 4 dpf; *n* = 16–32 larvae/batch, repeated at least twice. mRNA encodes predicted proteins p.A73_K105del and p.(T151Rfs*25) corresponding to Family 1 and Family 2, respectively. mRNA coding for p.Q56L is present in public databases (rs753489644; gnomAD browser), and scores as a benign variant in this assay. In panels **B** and **D**, ends of the whiskers are set at 1.5 times the interquartile range (IQR) above the third quartile and below the first quartile, respectively. Black dots, minimum and maximum outliers; *P < 0.05; **P < 0.01; **** *P* < 0.0001; (Kruskal-Wallis with Dunn’s multiple comparisons test).

We asked whether *rpl17* F0 mutants display anemia due to erythrocyte hypoplasia using zebrafish embryos harboring the *gata1:dsRed* transgene (46), a marker for erythroid progenitors expressed by 30 hours postfertilization (47). We conducted fluorescent live imaging of laterally positioned larval batches at 3 days postfertilization (dpf) and counted cells within a consistent region of interest. *rpl17* F0s displayed a significant reduction in the median number of dsRed^+^ cells compared with controls or batches injected with sgRNA alone (median reduction of 46% and 38%, respectively; Figure 3, A and B and Supplemental Table 4). Next, we designed a splice-blocking morpholino (sb-MO) targeting the *rpl17* exon 3 splice donor site (Supplemental Figure 4A). After demonstrating that the sb-MO results in exclusion of *rpl17* exon 3, (Supplemental Figure 4, B and C), we injected increasing doses (1, 2, or 4 ng) into *gata1:dsRed* transgenic embryos and repeated our assessment of erythroid cell abundance. We observed a significant dearth of dsRed^+^ cells in morphants injected with 4 ng sb-MO, similar to *rpl17* F0s (mean reduction of 47% versus controls; Supplemental Figure 4D and Supplemental Table 4). Coinjection of capped WT human *RPL17* mRNA significantly rescued this defect, restoring erythroid cell levels near to controls (Figure 3B and Supplemental Table 4). Together, our data indicate that disruption of *rpl17* in zebrafish alters erythropoiesis and supports a direct involvement of *RPL17* mutations in anemia.

Congenital malformations, including face and digit anomalies, exhibit variable expression in DBA (2), but were evident in family 1, particularly the index case (1-IV-2; Table 1, Figure 1, A and B, Supplemental Figure 1, A and B, and Supplemental Case Reports). We assessed each of the F0 mutant and morphant models using the *–1.4col1a1:egfp* transgenic line (48), which permits visualization of cartilage. We acquired live fluorescent images of larvae at 4 dpf and noted a similar cartilage patterning defect between *rpl17* F0s and morphants; this included an anterior-posterior shortening of the pharyngeal skeleton and hypoplasia of the branchial arches (Figure 3C). We measured the angle of the ceratohyal (ch) cartilage on ventral images and observed that sgRNA and Cas9-injected larvae, but not sgRNA-alone injected larvae, displayed a significantly broader ch angle than controls. These findings were recapitulated in experiments where larvae injected with 4 ng sb-MO exhibited a significant increase in the ch angle. Importantly, the morphant phenotype was improved significantly in larvae coinjected with capped human WT mRNA and sb-MO (Figure 3, C and D, Supplemental Figure 4E, and Supplemental Table 5).

To assess the potential pathogenicity of the *RPL17* variants (Figure 1, D and E), we conducted in vivo complementation studies, an approach that we have used previously (49–53). We generated capped human *RPL17* mRNA encoding the p.A73_K105del and p.(T151Rfs*25) variants in families 1 and 2, respectively, and the p.Q56L change identified by targeted resequencing. We compared the ability of each variant mRNA to rescue the ch angle defect versus WT mRNA in the presence of 4 ng sb-MO at 4 dpf. In replicate experiments, we were unable to detect significant differences between variant rescue and WT rescue of MO phenotype using a conservative multiple comparisons test, likely due to the dynamic range of the assay. However, pairwise comparisons with a Student’s *t* test showed that p.A73_K105del is a functional null; p.(T151Rfs*25) is a hypomorph; and p.Q56L is benign in this assay (Figure 3D; Supplemental Table 5). Variant *RPL17* mRNA did not result in craniofacial phenotypes that were different from WT mRNA (Supplemental Figure 5; Supplemental Table 5). In summary, our in vivo zebrafish models recapitulate the hematopoietic and cartilage phenotypes in individuals with *RPL17* variants and provide molecular evidence that the variants identified in families 1 and 2 are pathogenic.

### Ribosomal assembly studies show defects in rRNA biogenesis.

**Figure 4:**
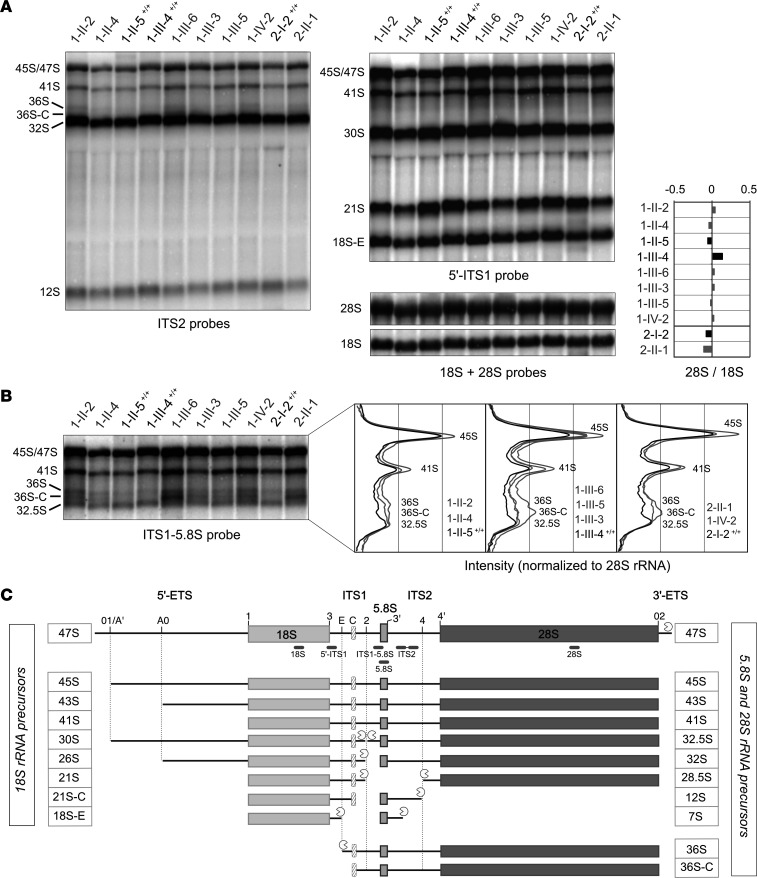
Analysis of ribosome synthesis show defects in rRNA maturation. (**A**) Total RNAs extracted from LCLs of DBA cases (gray) or unaffected individuals (black) were analyzed by Northern blot with probes ITS2, 5′-ITS1, 18S, and 28S (positions of the probes in panel **C**). The ratios of 28S to 18S rRNAs were quantified, normalized to the mean value obtained for controls, and are displayed as log values. (**B**) Detection of the cryptic pre-rRNA species 36S, 36S-C, and 32.5S with the ITS1-5.8S probe. The intensity profiles shown for the ITS1-5.8S probe were normalized relative to the levels of 28S rRNA. (**C**) Schematic representation of the pre-rRNAs derived from the 47S primary ribosomal transcript in human cells by endonucleolytic cleavage (horizontal lines) and exonucleolytic processing (Pacman). Impairment of cleavage at site 2 leads to accumulation of the 36S and 36S-C cryptic precursors by direct ITS1 cleavage at site E. Domain C corresponds to a highly conserved domain in ITS1 that blocks exonuclease progression. The positions of the Northern blot probes are indicated below the 47S pre-rRNA and their sequences are listed in Supplemental Table 8.

**Figure 5:**
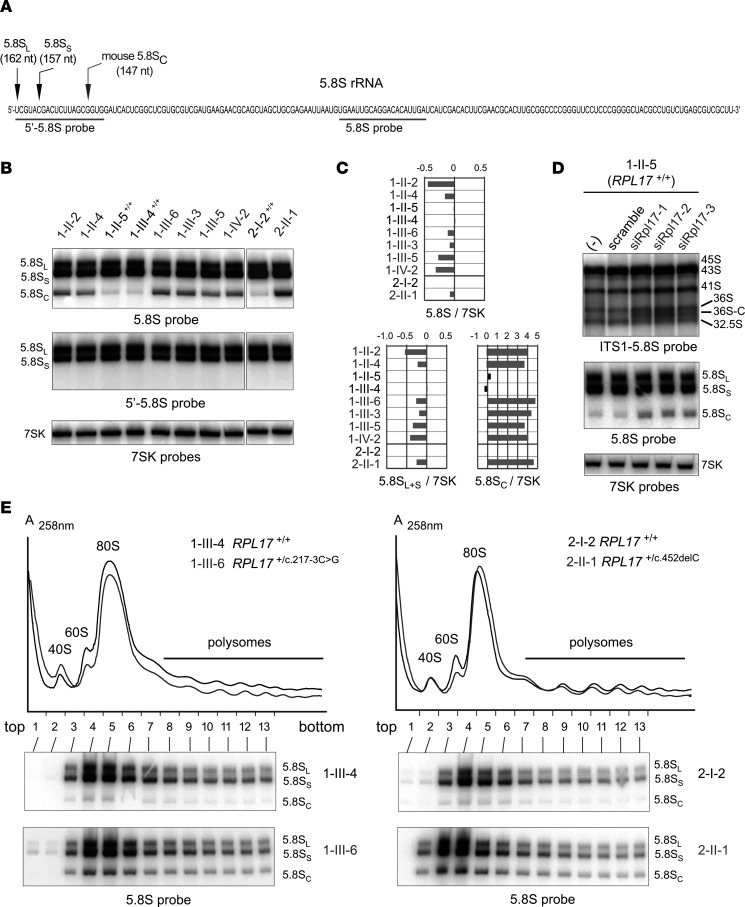
A very short form of 5.8S rRNA contributes to ribosome heterogeneity in *RPL17*^+/mut^ cells. (**A**) Sequence of the human 5.8S rRNA with the 2 canonical 5′ ends and that of mouse 5.8S_C_ rRNA previously determined (54), with the positions of the probes used in this figure. (**B**) 5.8S rRNA species separated on a 6% polyacrylamide gel (bottom) were identified with the 5.8S probe hybridizing to the core of the 5.8S rRNA, or with the 5′-5.8S probe complementary to the 5′-end of the 5.8S (**A**). The 7SK RNA was used as a loading control. (**C**) The ratios of the 5.8S rRNA species to 7SK snRNA (**B**) were quantified. Values were normalized to the mean value obtained for controls and are displayed as log_2_ values. (**D**) LCLs from unaffected individual 1-II-5 were treated with 3 siRNAs targeting *RPL17* mRNA. Total RNA profiles analyzed on agarose (top) or polyacrylamide gels (bottom) were compared to those of untreated cells (–) or cells treated with a scramble siRNA. (**E**) Cytoplasmic fractions extracted from control (black) or case (gray) lymphoblastoid cell lines were analyzed by ultracentrifugation on 10%–50% sucrose gradients. Total RNAs from the gradient fractions were extracted and analyzed by Northern blot with the 5.8S probe.

Analysis of total RNA from LCLs (family 1, 6 cases; family 2, 1 case) by Northern blot revealed impaired production of both the large and the small subunit in affected individuals (Figure 4, A and B). Early precursors (45S, 43S, and 41S), together with the cryptic 36S and 36S-C pre-rRNAs, were more prominent compared with unaffected family members (Figure 4B, ITS1-5.8S probe and insets). This pattern reflects a defective cleavage at site 2 and direct cleavage of the long precursors at site E, which releases the 18S-E and 36S pre-rRNAs (Figure 4C). The most striking phenotype, however, concerned the production of the 5.8S rRNA (Figure 5). In addition to the 2 canonical forms of 5.8S rRNAs (5.8S_L_ and 5.8S_S_, which differ by 5 nt in their 5′ extremity), we observed a shorter form termed 5.8S_C_ (Figure 5, A and B). This species was not detected by a probe hybridizing in the 5′ end of 5.8S_L_ rRNA (Figure 5B), indicating that it corresponds to a 5.8S rRNA variant truncated in its 5′ extremity. The 5.8S_C_ rRNA was also detected in LCLs from control individuals, but represented no more than 1% of total 5.8S rRNAs (samples 1-II-5, 1-III-4 and 2-I-2; Figure 5B). In contrast, 5.8S_C_ consisted of 8% to 21% of 5.8S rRNAs in affected case-derived cells. A similar form of 5.8S rRNA was shown previously to accumulate in mouse and human cells depleted of RPL17 (54), albeit in reduced quantities. The 5′ extremity of this species in mouse corresponds to the 16th nucleotide of 5.8S_L_ rRNA (Figure 5A), which is in agreement with the relative migration of 5.8S_C_ that we observe (5.8S probe in Figure 4, B and C; see Figure 5B for a description of 5.8S species).

Consistent with these results, treatment of LCLs with siRNAs targeting *RPL17* mRNA increased the proportion of 5.8S_C_ species to 5 %, compared with 0.8% in control cells (Figure 5D). *RPL17* knockdown also led to accumulation of the 45S, 43S, 41S, 36S, and 36S-C pre-rRNAs, reproducing the pattern observed in patient cells. Interestingly, *RPL17* knockdown in HeLa cells also affected cleavage at site 2, with an increase of 36S and 36S_C_ pre-rRNAs at the expense of 32.5S precursors (Supplemental Figure 6A). Impaired 28S and 5.8S rRNA production translated into lower 28S/18S and 5.8S_L+S_/7SK ratios (Supplemental Figure 6, B and C). However, the level of 5.8S_C_ rRNAs remained low (less than 1% of total 5.8S rRNA species), similar to previous observations in mouse 3T3 cells (54).

Separation of cytoplasmic ribosomal subunits on a sucrose gradient showed a decrease of the 60S peak relative to the 40S peak in cells from affected individuals in both families, confirming that large subunit production was affected (Figure 5). By contrast, the monosome (80S) and the polysome profiles remained similar to those observed in controls. Importantly, ribosomes containing the 5.8S_C_ rRNA were detected in polysomes, representing approximately 12% and 15% of 5.8S rRNAs detected in these fractions for individuals 1-III-6 and 2-II-1, respectively (Figure 5A). This indicates that 60S particles containing 5.8S_C_ rRNA are recruited efficiently into polysomes and suggests that they are active in translation. Relative quantification of RPs RPL17, RPL3, RPL5, and RPS19 in whole cell extracts by Western blot indicated that RPL17 stoichiometry was not modified in ribosomes of the patients’ LCLs (Supplemental Figure 7), consistent with the essential role of RPL17 in 60S subunit formation already reported in yeast and mice. Along this line, sucrose gradient analyses showed that ribosome production in HeLa cells was severely impaired upon *RPL17* knockdown with siRNAs; the 60S and 80S peaks were strongly decreased, while polysome peaks showed halfmers, i.e., translated mRNAs with an additional 40S subunit paused at the translation initiation codon awaiting binding of a 60S subunit (Supplemental Figure 6D). We conclude from these data that human RPL17 binding to the 5.8S sequence in pre-rRNAs drives cleavage in the ITS1 at site 2 and controls the proper maturation of the 5.8S rRNA 5′ end.

### RPL17 variants drive a translational response similar to RPS19 variants.

**Figure 6:**
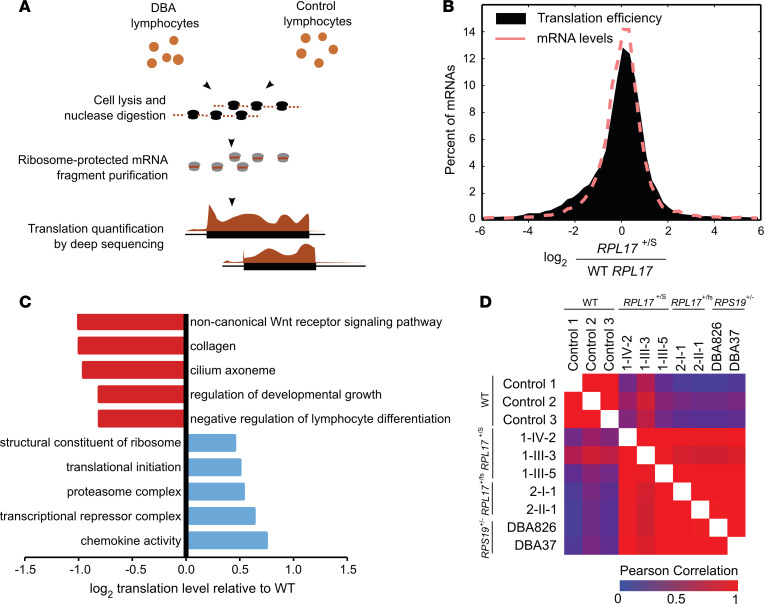
A specific translational response in LCLs of patients with DBA. (**A**) Experimental schematic. Cells from DBA cases or from healthy controls were lysed and treated with nuclease to degrade mRNA not protected by ribosomes. Ribosome-protected mRNA was then purified and deep sequenced to quantify translational activity. (**B**) Histogram showing the changes in ribosome density (ribosome profiling read density divided by RNA-Seq read density) in DBA cells relative to controls. The change in mRNA levels is also shown. All DBA cells carrying the *RPL17* c.217-3C>G genotype are included. (**C**) Gene ontologies enriched in mRNAs that have suppressed or enhanced translation. The log_2_ change represents the mean change in total translation across all DBA cells, with *P* values calculated by bootstrapping. (**D**) Relationships in translational activity between WT and DBA cells. Similarities are calculated by Pearson’s correlation coefficient.

Given the specific increase in 5.8S_c_-containing 60S particles observed with *RPL17* variants, we investigated whether these atypical ribosomal particles might have mRNA-specific effects on translation. To quantify translation at the level of individual transcripts, we analyzed LCLs from mutation-bearing individuals in each family by ribosome footprinting (Figure 6A). We performed RNA-Seq in parallel, allowing definition of the ribosome density (ribosomes per mRNA) for each gene and compared these results to LCLs from healthy individuals.

Analysis of LCLs from 3 individuals heterozygous for *RPL17* c.217-3C>G (1-IV-2, 1-III-3, and 1-III-5) showed specific translational changes, as evidenced by the distribution of mRNA levels and concomitant shift in ribosome density compared with matched controls (Figure 6B). Differences in mRNA levels were modest, as indicated by a tightly distributed histogram. We observed a subset of mRNAs that had markedly decreased ribosome footprint densities, suggesting reduced translational efficiency (Figure 6B). Using a cutoff of 4-fold change and a *P* value of less-than 0.05 by Student’s *t* test, there were 376 mRNAs that showed significantly suppressed ribosome density compared with 89 with enhanced ribosome density (Supplemental Table 6). Gene ontology (GO) analysis identified functional categories of transcripts that were impacted by these translational changes (Figure 6C and Supplemental Table 7). This included the ‘noncanonical Wnt receptor signaling pathway’ GO, GOs related to cilia, as well as several GOs related to development, particularly of the lymphocyte lineage (Figure 6C and Supplemental Table 7). Conversely, there were also some GOs that had statistically significant increases in ribosome density, although the magnitude of these increases was much smaller than suppressed GOs (Supplemental Table 7). This gene set was highly enriched in many components of the translational machinery; translation initiation and elongation factors as well as RPs all experienced modest, but significantly enhanced, translation.

To assess whether these translational changes were conserved across different DBA-associated mutations, we compared LCLs from family 1 (*n* = 3; *RPL17* c.217-3C>G) and family 2 (*n* = 2; *RPL17* c.452delC) with LCLs from 2 unrelated cases with a previously reported (61) *RPS19* variant. To test for variations in the translation profiles between cell lines, the level of translation for each gene in each cell line was plotted, and *r* values derived. We observed a pattern in which the cell lines derived from controls were similar to one another, while all the DBA-mutation bearing cell lines were similar to one another, yet distinct from the controls (Figure 6D). Translational enhancement of mRNAs encoding RPs was consistently found in each of the 3 DBA groups. Total apparent translation of RPs (i.e., ribosome footprint densities) increased by, on average, approximately 50% (Supplemental Figure 8). The mRNAs, however, were mostly unchanged or decreased in abundance, as assessed by RNA-Seq (Supplemental Figure 8), which is consistent with a robust increase in the translation efficiency of these mRNAs in response to RP haploinsufficiency. This is in contrast with the decrease in RP synthesis reported in cells depleted of a RP by small hairpin RNAs (shRNAs) (55, 56) and may reflect a long-term response to defects in ribosome biogenesis in the context of haploinsufficiency, as opposed to a short-term response to a strong RP depletion triggered by shRNAs. Overall, these results indicate that diverse DBA-related variants, including in different RP genes, drive a consistent translational response that may contribute to the disease phenotypes.

## Discussion

### Haploinsufficiency of RPL17 is associated with DBA.

Here, using multi-tiered clinical, genetic, and biochemical studies involving in vivo and in vitro models, to our knowledge, we have added to the variant spectrum of DBA with the discovery of *RPL17* as a rare causal gene. The 2 pedigrees we identified harbor pathogenic *RPL17* variants validated by in vivo complementation assay in zebrafish models. We observe extensive inter- and intrafamilial phenotypic variability and incomplete penetrance that is not uncommon in DBA (2). On the severe end of the phenotype spectrum, the family 1 index case (1-IV-2) is reminiscent of acrofacial or mandibulofacial dysostosis typified by Treacher-Collins, Nager, or Miller syndromes (57–59). Alternatively, the father of the family 2 proband (2-I-1) is reportedly healthy, but harbors the same variant as his affected son. Consistent with a broad range of autosomal dominant disorders in humans (e.g., retinitis pigmentosa caused by the splicing factor *PRPF31* (60)), such discrepancies can likely be explained by effects in cis (expression level of WT *RPL17* mRNA in variant carriers), or effects in trans (mutational burden, protective alleles, or variable expression of functionally related transcripts).

The medical history of the affected individuals in family 1 also shows variability of the hematopoietic condition; only 4 out of the 8 individuals bearing the *RPL17* variant displayed anemia, and only 1 faced a severe episode (1-III-3). In these cases, as well as in the affected individual of family 2, anemia remained transient and manifested later than in the majority of patients with DBA (usually before 3 months of age). This is consistent with a recent report suggesting that the hematopoietic phenotype is less severe, on average, in patients with DBA with variants of *RPL* genes relative to *RPS* genes (61). The erythroid cultures performed here clearly showed a defect in erythroid progenitor proliferation, but suggested that differentiation is maintained similar to erythroid cells from patients with *RPS19* variants in a similar assay (25). Finally, the patients with *RPL17* variants displayed other hematopoietic disorders, sometimes in parallel to anemia, including leukopenia and thrombocytopenia, which further illustrates the need to extend the definition of DBA to a broader range of conditions than pure red cell aplasia.

### RPL17 haploinsufficiency generates 60S particles containing 5.8S_C_ rRNA.

Our results reveal that LCLs derived from DBA cases with RPL17 haploinsufficiency contain a substantial proportion of a very short form of 5.8S rRNA (5.8S_C_). This species is also present at very low levels under homeostatic conditions in control LCLs. Studies in mouse cells have suggested that the 5′ end of the 5.8S_C_ species is tailored by the 5′ exonuclease XRN2 on the 32S precursor, as it is for the 2 canonical 5.8S_L_ and 5.8S_S_ rRNAs (54).

In *Saccharomyces cerevisiae*, the 5′ extremity of the 5.8S_S_ rRNA is also formed by the 5′-exonuclease Rat1, the homolog of XRN2 (62), while the 5.8S_L_ form is produced by endonucleolytic cleavage (63). Biochemical and structural studies in yeast have clearly established that RPL17 controls the 5′ exonucleolytic processing of the 5.8S_S_ rRNA 3′ end (64–67). As shown by cryoelectron microscopy, RPL17 becomes stably associated with the pre-60S subunit upon release of ribosome biogenesis factors Noc1-Noc2, whose departure exposes the 5.8S rRNA 5′ end to exonucleolytic processing (67). RPL17 directly contacts the 5.8S rRNA 5′ end and is likely to form a physical barrier that limits exonucleolytic trimming. Accordingly, RPL17 depletion in yeast allows Rat1 to trim the 5.8S rRNA downstream of the 5.8S_S_ extremity (65, 66). These data suggest that the kinetics of RPL17 stable association with pre-60S particles is important to control 5′ exonucleolytic processing.

Based on these data, we propose that lower concentrations of neosynthesized RPL17 in haploinsufficient cells affect the kinetics of RPL17 incorporation into nascent 60S subunits and thereby allow 5.8S rRNA trimming by XRN2 beyond the 5.8S_S_ 5′ end. We found no significant global change in RPL17 stoichiometry relative to other RPs in patient LCLs (Supplemental Figure 7), which rules out the mere absence of RPL17 in mature ribosomes. Moreover, stringent knockdown of *RPL17* expression with siRNAs in HeLa cells performed in this work or in other human or mouse cell lines (54) severely affected pre-rRNA processing but did not result in marked accumulation of 5.8S_C_ RNA. This likely reflects rapid turnover of pre-60S particles missing RPL17 (66), as attested by the deficit of cytoplasmic 60S subunits and the presence of halfmers in polysomes (Supplemental Figure 6). In contrast, we did observe accumulation of 5.8S_c_ rRNA in LCLs treated with *RPL17* siRNAs. We hypothesize that *RPL17* knockdown was only partial in LCLs, which may better mimic haploinsufficiency and reproduce the kinetic delay of RPL17 association with the pre-60S particles discussed above; this would then allow formation of the 5.8S_c_ RNA without impeding RPL17 incorporation in a fraction of pre-60S particles.

### Impact on translation.

Our findings represent a unique example of a marked qualitative change in rRNA structure in DBA. The 5′-end of the 5.8S rRNA is located in the peptide exit tunnel (PET) of the 60S subunit, where it may act as a sensor of the nascent peptide. RPL17 directly interacts with RNA helix 2 in the large subunit, which results from the hybridization of the 5.8S rRNA 5′-end with a segment of the 28S rRNA. By destabilizing helix 2, further shortening of this 5.8S extremity may affect the conformation of RPL17 in the PET (54) and thereby alter progression of the nascent peptide or impair the binding of chaperones involved in translation (68). Along this line, a specific impact on synthesis and stability of proteins were recently observed when RPL39, another PET protein, is replaced by its paralog RPL39L (69).Despite this remarkable change in ribosome structure, the phenotype observed in the patients reported here is highly reminiscent of other patients with DBA with pathogenic variants in RPs unrelated to the exit tunnel. Further, the Ribo-Seq experiments show a common translational response in LCLs bearing *RPL17* and *RPS19* variants, echoing results reporting similar translational profiles in primary human hematopoietic stem and progenitor cells (HSPCs) depleted of RPs RPL5 and RPS19 (55) or in mouse erythroblasts depleted of RPL11 and RPS19 (56) using shRNAs. Of note, while LCLs are not a model directly relevant for DBA pathophysiology, the cohort of mRNAs whose translation was suppressed in the mutant background was enriched in genes linked to tissue development, which is often affected in DBA. Alterations of the translation of developmental genes may be part of the pathophysiological mechanisms in various tissues, as proposed more specifically for erythroblasts in which translation of the *GATA1* mRNA was shown to be affected (55).

These findings reinforce the hypothesis that the primary determinants of DBA are the decreased rate of ribosome production (5) and the stress responses triggered by defects in ribosome biogenesis. Further work is required to untangle the relative contribution of translation alteration and stress response in this disease; in addition, it remains to be explained why other ribosomopathies that affect ribosome production do not show the erythroid disorder commonly observed in DBA.

## Methods

### Sex as a biological variable.

This study involved male and female individuals. Sex was not considered as a biological variable.

### Human cohorts and sample ascertainment and ethics statement.

The cohort of 55 individuals with DBA utilized for targeted resequencing of *RPL17* are registered in the French DBA registry (CNIL acceptance N°911387, CCTIRS-N°11.295, 12/05/2011); DBA was diagnosed according to established criteria (2).

### Genetic analysis (exome sequencing and targeted resequencing).

For family 1, ES was conducted on 3 affected individuals (1-III-3, 1-III-6, and 1-IV-2). Genomic DNA was fragmented by Covaris to generate fragments between 150 to 200 bp. Adapters were ligated to both ends of the resulting fragments, adapter-ligated templates were purified by the Agencourt AMPure SPRI beads and fragments with insert size of approximately 250 bp were excised. Extracted DNA was amplified by ligation-mediated PCR (LM-PCR), purified, and hybridized to the SureSelect Biotinylated RNA Library (Agilent) for enrichment. Each captured library was sequenced on a Hiseq2000 platform. Raw image files were processed by Illumina base calling Software 1.7 for base calling with default parameters, and the sequences of each individual were generated as 90 bp pair-end reads. Exome data were processed as described previously (70). Two patients of family 1 were reported separately in an overview of DBA in Switzerland (71).

For family 2, ES was performed on the index case (2-II-1) and his healthy parents (2-I-1 and 2-I-2). Genomic DNA was prepared using Kapa Hyper library prep, followed by exome enrichment using Nextera. Sequencing was performed using an Illumina HiSeq 2500 instrument. Bidirectional sequence was assembled, aligned to reference gene sequences based on human genome build GRCh37/UCSC hg19, and analyzed using the custom-developed software RUNES and VIKING (34, 72), variants were filtered to 1% MAF in our local variant database, then prioritized by the ACMG categorization (73), OMIM identity, and phenotypic assessment. Integrative Genomic Viewer software version 2.3.8 (IGV; Broad Institute, Cambridge, Massachusetts, USA) was used to view alignments. All potential inheritance patterns were investigated. Homozygous, heterozygous, and compound heterozygous variants with an allele frequency less than 1% in our local database of more than 5,000 samples were analyzed, in addition to variants unique to the patient (de novo). In each of families 1 and 2, *RPL17* variants were confirmed in samples from all available family members using BigDye terminator chemistry and bidirectional capillary sequencing (Applied Biosystems) according to standard methods. Finally, we sequenced the coding regions and splice junctions of *RPL17* in a cohort of 55 unrelated individuals fulfilling clinical diagnostic criteria for DBA using bidirectional Sanger sequencing. Primers are available in Supplemental Table 8.

### Lymphoblastoid cell culture, RNA isolation, and RT-PCR.

We separated B cell lymphocytes from whole blood, transformed them with EBV according to standard procedures, and cells were cultured in RPMI-1640 supplemented with 10% FBS. To evaluate the effect of the c.217-3C>G variant, we harvested cells in Trizol (Thermo Fisher Scientific) and conducted oligodT primed reverse transcription (RT) with the QuantiTect Reverse Transcription kit (Qiagen). The region flanking the *RPL17* splice acceptor was PCR amplified, migrated on an agarose gel, and gel-purified products (QIAquick Gel Extraction Kit; Qiagen) were subjected to direct bidirectional sequencing. Primer sequences are available in Supplemental Table 8.

### Culture of human primary cells.

CD34^+^ cells from peripheral blood were isolated by immunomagnetic technique using a manual column (Miltenyi Biotec). Purified CD34^+^ cells were cultured according to the erythroid culture protocol described previously (29) for cell synchronization using 3% AB serum (Sigma-Aldrich), 2% human peripheral blood plasma (Stem Cell Technologies), 10 μg/mL insulin (Sigma-Aldrich), 3 IU/mL heparin (Sigma-Aldrich), 200 mg/mL holo-transferrin (Sigma-Aldrich), 10 ng/mL stem cell factor (SCF) (Miltenyi Biotec), 1 ng/mL IL3 (Stem Cell Technologies), 3 IU/mL erythropoietin (EPO) and 1% penicillin/streptomycin. This media was used from D0 to D6, then deprived from IL3 from D7 to D10. From D11 to D14, SCF was removed and EPO was used at 0.1 IU/mL. All cell cultures were maintained at 37°C with 5% CO_2_. Viable cells were counted in triplicate using the trypan blue dye exclusion test as a function of time in culture.

### Erythroid proliferation, differentiation, and apoptosis studies.

Erythroid proliferation and differentiation were evaluated by fluorescence-activated cell sorting (FACS) on an InFlux instrument (BD Biosciences). 10 × 10^3^ to 5 × 10^6^ human primitive cultured erythroid cells were immunophenotyped from D5 to D14 using several antibodies, including PC7 or PE conjugated CD34 (Beckman Coulter; ref. A51077 and IM1871U, respectively); APC conjugated CD36 (BD Biosciences; ref. 550956); PE/Cy7 conjugated IL-3R (Miltenyi Biotec; clone BVD3-1F9); Glycophorin A (CD235) (Life Technologies; ref. 17-9987-42); APC conjugated Band 3 (provided by Mohandas Narla’s lab, New York Blood Center, New York, New York, USA). To study apoptosis, cells were stained with PerCP-Cy5.5–conjugated Annexin V (BD Biosciences, ref. 561431) and with DAPI (Sigma-Aldrich) according to the manufacturer’s protocol, and separated by FACS on an InFlux flow cytometer (BD Biosciences). Data were analyzed using Kaluza software (Beckman Coulter). Isotype controls were obtained from Becton Dickinson.

### RPL17 immunoblotting.

LCLs were harvested, centrifuged, and washed twice in PBS. Cell pellets were lysed in 250–500 μl of lysis buffer (50 mM Tris-HCl, pH 7.4, 1 mM EDTA, 1% Triton X-100, 200 mM NaCl, 1 × Complete EDTA-Free protease inhibitor cocktail (Roche Diagnostics)) on ice for 30 minutes under gentle agitation. The extracts were then cleared by centrifugation for 15 minutes at 13,000*g* at 4°C. Eleven micrograms of proteins were mixed with reducing loading buffer, heated for 10 minutes at 90°C, and separated on 8%–12% SDS-PAGE gels (Invitrogen). Proteins were transferred to nitrocellulose membranes (GE Healthcare) in NuPage transfer buffer, 20% ethanol by wet transfer for 3 hours at 4°C with a tension of 90V. Membranes were stained with Ponceau S (Sigma-Aldrich) to assess transfer efficiency and blocked in PBS, 0.05% Tween-20, 5% nonfat dry milk for 1 hour at room temperature.

RPs were detected on the blots with rabbit polyclonal antisera directed against mammalian RPL17 (GeneTex, ref. GTX101831, diluted 1:1,000), RPL5 (GeneTex, ref. GTX101821, diluted 1:1,000), and RPL3 (GeneTex, ref. GTX114725, diluted 1:1,000). The blots were also probed with a custom-made rabbit polyclonal antiserum against the human RPS19 protein and a commercial mouse monoclonal antibody against actin (Sigma-Aldrich, ref. A4700, diluted 1:1,000). HRP-conjugated secondary antibodies (Promega, Anti-mouse ref. W402B, Anti-rabbit ref. W401B) were used at a dilution of 1:5,000. Chemiluminescent detection with ECL reagent (Thermo Fisher Scientific) was performed on a Chemidoc Imaging system (Bio-Rad), and signals were quantified using Image J (74).

### CRISPR/Cas9 genome editing of rpl17.

Experiments were performed according to protocols approved by the Duke University IACUC. Zebrafish embryos were obtained from natural matings of heterozygous *gata1:dsRed* adults maintained on an EkkWill (EK) background (46) with heterozygous *–1.4col1a1:egfp* transgenic adults maintained on an AB background (48). We targeted the zebrafish *rpl17* locus a with a guide RNA (gRNA) complementary to the target 5′-GGCAAGGGGAGCTCATGTAGGGG-3′, which was designed using CHOPCHOP (75). gRNA was transcribed in vitro using the GeneArt precision gRNA synthesis kit (Thermo Fisher Scientific) according to manufacturer’s instructions (template oligos are available in Supplemental Table 8). To determine gRNA efficiency in founder (F0) mutants, we injected 100 pg gRNA with 200 pg Cas9 protein (PNA Bio) into the cell of single-cell–stage embryos. We harvested embryos at 2 dpf for genomic DNA extraction, PCR-amplification of the targeted region (Supplemental Table 8), and heteroduplex analysis as described (53). To estimate mosaicism in *rpl17* F0s, we gel purified PCR products (Qiagen) and cloned them into a TOPO-TA vector (Thermo Fisher Scientific); *n* = 2 controls and 6 F0 mutants. We extracted the plasmids from 12 colonies per embryo and Sanger sequenced them according to standard procedures. Phenotyping assays were conducted with 100 pg gRNA with or without 200 pg Cas9 protein.

### Zebrafish in vivo complementation studies.

To suppress endogenous *rpl17,* we designed and synthesized an sb-MO (5′-AGAGTTAAATCTTACCTTGAAGTGA-3’; GeneTools). To determine MO efficiency, we injected embryo batches (*n* = 20/batch) with increasing doses of MO (3, 6, and 9 ng per embryo; 1 nL per injection), and harvested embryos in Trizol for total RNA isolation (Thermo Fisher Scientific). We conducted RT-PCR as described for lymphoblastoid cells above and characterized the resulting PCR products using bidirectional sequencing of purified PCR products. To generate capped human *RPL17* mRNA for in vivo complementation studies, we used LR clonase II (Thermo Fisher Scientific) to recombine a WT *RPL17* ORF entry clone (IOH3517; Thermo Fisher Scientific) into a Gateway-ready pCS2+ destination (GWdest) construct. To generate mutant constructs, we used either site-directed mutagenesis as described (76) or we PCR-amplified the *RPL17* ORF from mutation carriers of family 1, cloned the resulting product into the pCR8/TOPO vector, and recombined it into the pCS2+GWdest vector. Subsequent to linearization, sequence-confirmed WT and mutant constructs were transcribed in vitro with the mMessage mMachine SP6 Transcription Kit (Thermo Fisher Scientific). Unless otherwise noted, in vivo complementation experiments were conducted by injecting 4 ng MO and/or 100 pg RNA into the yolk of embryos at the 1-to-4 cell stage with the investigator masked to the injection cocktail.

### Zebrafish phenotyping assays.

Larvae were maintained according to standard procedures at 28.5˚C until the appropriate stage for phenotyping; they were then anesthetized with Tricaine (MS-222) at either 3 dpf (erythrocyte quantification) or 4 dpf (cartilage patterning) before live imaging. We acquired lateral images of larvae harboring the *gata1:dsRed* transgene using an AZ100 fluorescent microscope (Nikon) equipped with a Digital Sight monochromatic camera (Nikon) and NIS Elements AR software (Nikon). Erythrocytes in a consistently defined area starting from the cloaca and spanning the width of 2 somites were quantified using the image-based tool for counting nuclei (ITCN) plugin of ImageJ (NIH). To assess cartilage structures in larvae expressing the *–1.4col1a1:egfp* transgene, we positioned and imaged the ventral aspect of larvae using the Vertebrate Automated Screening Technology (VAST) platform (Union Biometrica) mounted on an AxioScope A1 microscope (Zeiss) equipped with an Axiocam 503 monochromatic camera and Zen Pro 2012 software (Zeiss), essentially as described (50, 53); we determined the ceratohyal cartilage angle using ImageJ. A Kruskal-Wallis with Dunn’s multiple comparisons test was used to identify statistical differences between multiple conditions within an experiment. All pairwise comparisons to determine statistically significant differences were calculated using a Student’s *t* test (2-tailed, unpaired).

### HeLa cell culture and RPL17 knockdown.

HeLa cells (ATCC) were cultured in DMEM (Gibco, Thermo Fisher Scientific) supplemented with 10% FBS and 1 mM sodium pyruvate (Sigma-Aldrich). Three different 21-mer siRNAs (Eurogentec), the efficiency of which were verified by qPCR, were used to knock down expression of human *RPL17* mRNAs in HeLa cells (Supplemental Table 8). The siRNA solution (500 nM final concentration) was added to the cell suspension (1 × 10^7^ cells in 200 μL of Na phosphate buffer, pH 7.25, 250 mM sucrose, 1 mM MgCl_2_) incubated on ice. Electro-transformation was performed with square pulses at 240 V with a Gene Pulser (Bio-Rad) as described (77). A scrambled siRNA (siRNA-negative control duplex; Eurogentec) was used as a control. Cells were then plated in DMEM containing FBS and grown at 37°C for 48 hours. The same protocol was applied to LCLs.

### Northern blot of pre-rRNAs.

For total RNA isolation, 2 × 10^6^–5 × 10^6^ cells were collected by centrifugation at 300*g* and rinsed with cold PBS. The pellets were mixed with Tri reagent (Sigma-Aldrich) and extracted with chloroform. The aqueous phases were purified further by phenol/chloroform/isoamyl alcohol (25:24:1) and chloroform extractions (Sigma-Aldrich, prior to alcohol precipitation. A similar protocol using Trizol LS was applied to extract total RNAs from sucrose fractions. Total RNA samples (3 mg/lane) were dissolved in formamide and separated on a 1.1% agarose gel containing 1.2% formaldehyde and Tri/Tri buffer (30 mM triethanolamine, 30 mM tricine, pH 7.9). Analyses of 5.8S rRNAs were performed on 6% polyacrylamide gels (19:1) (Bio-Rad) in TBE buffer containing 7 M urea. RNAs were transferred to Hybond N^+^ nylon membrane (GE Healthcare) and cross-linked under UV light. Membrane prehybridization was performed at 45°C in 6 × SSC, 5 × Denhardt’s solution, 0.5% SDS, 0.9 mg/mL tRNA. The 5′-radiolabeled oligonucleotide probe was added after 1 hour and incubated overnight at 45°C. After washing twice for 10 minutes in 2 × SSC, 0.1% SDS and once in 1 × SSC, 0.1% SDS, the membrane was exposed to a PhosphorImager screen. Radioactive signals were revealed using a Typhoon Trio PhosphorImager (GE Healthcare) and quantified using the MultiGauge software. The probes used are listed in Supplemental Table 8.

### Polysome gradients.

Lymphoblastoid cells (or HeLa cells 48 hours after transfection) were treated for 10 minutes with 100 μg/mL cycloheximide (Sigma-Aldrich). After washing with PBS containing cycloheximide, cell pellets were mechanically disrupted with a Dounce homogenizer in buffer A (10 mM K-HEPES, pH 7.9, 1.5 mM MgCl_2_, 10 mM KCl, and 0.5 mM DTT) containing cycloheximide. The cytoplasmic fraction was recovered by centrifugation for 10 minutes at 1,000*g* and at 4°C and then clarified further by 2 successive centrifugations at 10,000*g*. A volume corresponding to 1 mg total proteins was loaded on 10%–50% (w/w) sucrose gradients, prepared with a Gradient Master former (BioComp Instruments). After centrifugation at 4°C and at 222,000*g* for 105 minutes in a SW41 rotor (Optima L100XP ultracentrifuge; Beckman Coulter), the fractions were collected at OD_254__nm_ with a Foxy Jr. gradient collector (Teledyne Isco).

### Ribosomal profiling and RNA-seq.

Ribosome profiling was performed essentially as described (78–80). For LCLs, 5 × 10^6^ cells were collected by centrifugation then lysed in 250 μL 200 mM KOAc, 15 mM MgCl_2_, 25 mM K-HEPES pH 7.2, 4 mM CaCl_2_, 2% dodecylmaltoside. For samples where RNA-Seq was performed, 50 μL of the lysate was set aside and RNA was extracted using GT/phenol (81). With the remaining lysate, the sample was diluted 1:1 with water, then micrococcal nuclease (Sigma-Aldrich) was added to a final concentration of 20 μg/mL. The sample was incubated for 30 minutes at 37°C. Ribosomes were then pelleted through a 500 mM sucrose cushion at 352,000*g* for 40 minutes in a TLA-100.2 (Beckman-Coulter). The resulting ribosome pellet was resuspended in 200 μL 50 mM NaCl, 50 mM K-HEPES pH 7.2, 5 mM EDTA, 0.5% SDS, 200 μg/mL proteinase K. RNA was extracted by phenol/chloroform, then treated with polynucleotide kinase (New England Biolabs). Ribosome footprints were isolated by polyacrylamide gel electrophoresis, and deep sequencing libraries were prepared using the NEBNext Small RNA Library Prep Set (New England Biolabs). RNA-Seq libraries were generated using the NEBNext Ultra Directional RNA Library Prep Kit for Illumina (New England Biolabs). All sequencing was performed using either Illumina HiSeq 2500 (for ribosome profiling) or Illumina Genome Analyzer (for RNA-Seq). Reads were mapped to the RefSeq transcriptome (RefSeq release 60), mapping to the longest transcript derived from each gene. Reads with more than 5 valid mapped positions were discarded and as many as 2 valid mappings were allowed. A 20 nt seed region was used. Following mapping, the position of each ribosome was defined by adding 14 nt to the start of each read. The abundance of ribosomes or of mRNA was determined by the number of coding sequence-mapped reads normalized by the length of the coding sequence and the size of each deep sequencing library. Ribosome density was defined as the number of ribosome foot printing read density divided by RNA-Seq read density. Statistical significance of differences in ribosome density was determined by Student’s *t* test. Gene ontology analysis was performed by bootstrapping, where the mean log_2_ difference in ribosome density was calculated then compared with random permutations to determine *P* value.

### Statistics.

Details of statistical tests are given in figure legends. Tests used included Kruskal-Wallis with Dunn’s multiple comparisons test (zebrafish morphometrics); unpaired Student’s *t* test, 2-sided (zebrafish morphometrics; differences in ribosome density); bootstrapping (ribosome profiling and RNAseq; Gene Ontology); unilateral Mann-Whitney *U* test (evaluation of RPL17 stoichiometry); and a *P* < 0.05 was considered significant.

### Study approval.

These studies were conducted in accordance with protocols approved by the Institutional Review Boards (or equivalent ethics review committees) at the University of Lausanne (Lausanne, Switzerland), Duke University Medical Center (IRB; Pro00022846; Durham, North Carolina, USA), the Lurie Children’s Hospital of Chicago (IRB 2019 2950; Chicago, Illinois, USA), Children’s Mercy Hospital (study # 11120514; Kansas City, Missouri, USA), and Hôpital Robert Debré (Paris, France). Informed written consent was obtained from all participants before study inclusion, including for the use of the photographs, and the record of informed consent has been retained. Zebrafish studies were conducted in accordance with protocols approved by the Institutional Animal Care and Use Committees (IACUC) at Duke University (A154-18-06; Durham, North Carolina, USA) and Northwestern University (IS00016405; Chicago, Illinois, USA).

### Data availability.

All *RPL17* variants have been deposited in the ClinVar database under accession numbers SCV005038631, SCV005038799, and SCV005038800. Processed transcriptome and ribosome profiling RNA sequencing data are available on Zenodo (https://doi.org/10.5281/zenodo.12571975). Data underlying quantitative analyses are provided in the Supporting Data Values file.

## Author contributions

FF, JF, JSB, RR, and ZAB performed clinical ascertainment, sampling and followup of DBA families. BRB, CR, CS, CY, MF, JZ, and PF performed genetic analyses on human samples. LDC and N Karboul conducted erythroid maturation studies. LDC provided DBA nongenotyped samples for RPL17 mutation screening. ALWK, EED, JRW, KAMF, LF, and N Katsanis generated and analyzed zebrafish models. MFOD, NML, JJ, and PEG performed ribosome biogenesis studies. CVN and DWR generated and analyzed ribosome profiling data. CS, CVN, DWR, EED, FF, MFOD, and PEG wrote the manuscript with input from all authors. CVN, EED, and PEG designed the study. FF, CS, and MFOD share the first-author position due to equivalent contributions. The order was chosen according to the first date of participation to the project.

## Supplementary Material

Supplemental data

Unedited blot and gel images

Supplemental table 6

Supplemental table 7

Supporting data values

## Figures and Tables

**Table 1:**
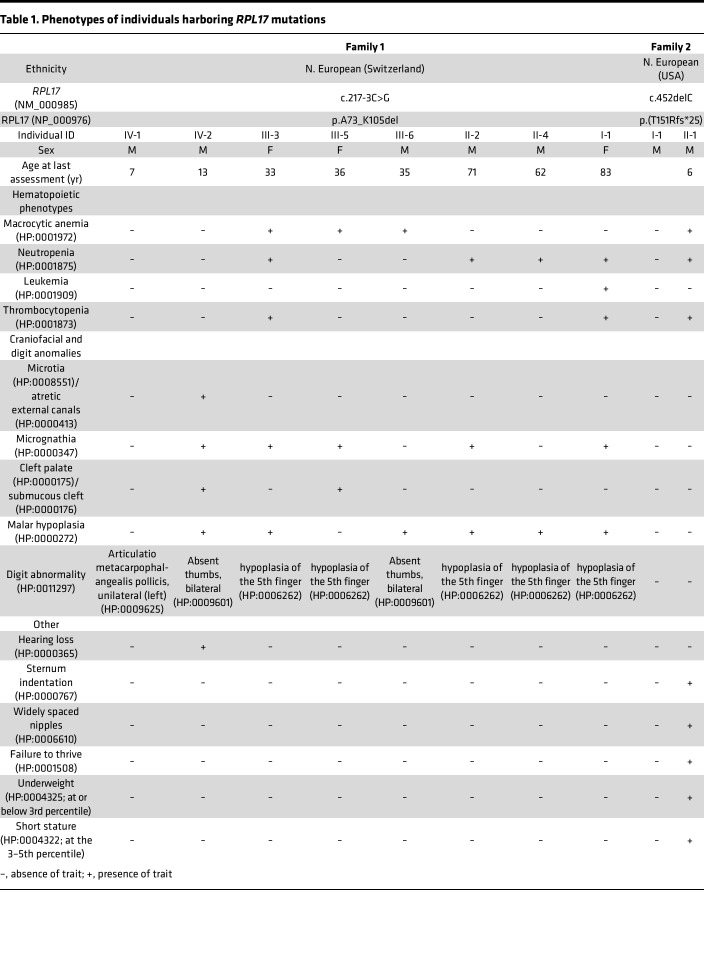
Phenotypes of individuals harboring *RPL17* mutations

## Version 1 08/01/2024

In-Press Preview

## Version 2 09/10/2024

Electronic publication
